# Retraction Note: In vitro and in vivo anti-colon cancer effects of Garcinia *mangostana xanthones* extract

**DOI:** 10.1186/s12906-022-03681-3

**Published:** 2022-07-23

**Authors:** Abdalrahim F. A. Aisha, Khalid M. Abu-Salah, Zhari Ismail, Amin Malik Shah Abdul Majid

**Affiliations:** 1 Department of Pharmacology, School of Pharmaceutical Sciences, Universiti Sains Malaysia, 11800 Minden, Pulau Penang Malaysia; 2 The Chair of Cancer Targeting and Treatment, Biochemistry Department and King Abdullah Institute for Nanotechnology, King Saud University, Riyadh, 11451 Saudi Arabia; 3 Department of Pharmaceutical Chemistry, School of Pharmaceutical Sciences, Universiti Sains Malaysia, 11800 Minden, Pulau Penang Malaysia; 4 Australian Institute for Nanotechnology and Bioengineering, University of Queensland, Queensland, 4072 Australia

**Retraction Note: BMC Complement Altern Med 12, 104 (2012)**

**https://doi.org/10.1186/1472-6882-12-104**

The Editor has retracted this article. Concerns have been raised about the welfare of the mice used in this study. The Editor has not been able to obtain clarification on the tumour volume exhibited by all of the mice or of the protocol followed for mice in which ulceration was observed. None of the authors has responded to correspondence from the Editor about this retraction.

